# Prolonged Neuropsychological Deficits, Central Nervous System Involvement, and Brain Stem Affection After COVID-19—A Case Series

**DOI:** 10.3389/fneur.2020.574004

**Published:** 2020-11-05

**Authors:** Stefan Jun Groiss, Carolin Balloff, Saskia Elben, Timo Brandenburger, Tomke Müttel, Detlef Kindgen-Milles, Christian Vollmer, Torsten Feldt, Anselm Kunstein, Björn-Erik Ole Jensen, Hans-Peter Hartung, Alfons Schnitzler, Philipp Albrecht

**Affiliations:** ^1^Department of Neurology, Medical Faculty, Heinrich-Heine University, Düsseldorf, Germany; ^2^Institute of Clinical Neuroscience and Medical Psychology, Medical Faculty, Heinrich-Heine University, Düsseldorf, Germany; ^3^Department of Anesthesiology, Medical Faculty, Heinrich-Heine University, Düsseldorf, Germany; ^4^Department of Gastroenterology, Hepatology and Infectious Diseases, Medical Faculty, Heinrich-Heine University, Düsseldorf, Germany

**Keywords:** COVID-19, SARS-CoV-2, electrophysiology, cognition, brain stem

## Abstract

**Objective:** The affection of both the peripheral (PNS) and central nervous system (CNS) by severe acute respiratory syndrome coronavirus 2 (SARS-CoV-2) has been assumed to play a direct role in the respiratory failure of patients with Corona virus disease 2019 (COVID-19) through affection of medullary cardiorespiratory centers resulting in neurological complications and sequelae.

**Methods:** We used a multimodal electrophysiological approach combined with neuropsychological investigations to study functional alteration of both the PNS and CNS in four patients with severe COVID-19.

**Results:** We found electrophysiological evidence for affection of both the PNS and CNS, and particularly affection of brain stem function. Furthermore, our neuropsychological investigations provide evidence of marked impairment of cognition independent of delirium, and outlasting the duration of acute infection with SARS-CoV-2.

**Conclusion:** This case series provides first direct electrophysiological evidence for functional brain stem involvement in COVID-19 patients without evident morphological changes supporting the notion of the brain stem contributing to respiratory failure and thus promoting severe courses of the disease. Moreover, sustained neuropsychological sequelae in these patients may be of particular psychosocial and possibly also economic relevance for society.

## Introduction

Corona virus disease 2019 (COVID-19) is caused by severe acute respiratory syndrome coronavirus 2 (SARS-CoV-2), which mainly affects the respiratory system (1). Although the vast majority of affected individuals only show mild to moderate symptoms, severe courses requiring intensive medical care have a high risk for mortality (1, 2). Neurological complications are common with more severe infections that disproportionately affect older patients and include impaired consciousness, stroke, and acute neuropathies (3–5). This is likely due to direct affection of both the central (CNS) and peripheral nervous system (PNS) by SARS-CoV-2 (5, 6). It is supposed that direct neuronal infection may be caused by affection of the angiotensin-converting enzyme 2 receptor (7). Studies on SARS-CoV-1 and middle east respiratory syndrome CoV (MERS-CoV) have suggested initial affection of the PNS, mostly of the olfactory nerve, leading to trans-synaptic transfer to the brain stem and spreading into the CNS (8–11). Other neural routes via the vagus nerve have also been described for other neurotropic viruses like influenza A (12, 13). Therefore, the neurotropic potential of SARS-CoV-2 may also play a direct role in the respiratory failure of patients through affection of medullary cardiorespiratory centers, such as the pre-Bötzinger complex, pneumotaxic center, or central chemoreceptor area (8, 10, 14).

Here, we present a series of four severely affected COVID-19 patients with first direct electrophysiological evidence for brain stem affection and neuropsychological sequelae.

## Methods

### Subjects

Four men with severe COVID-19 (59.5 ± 17.6 years) were investigated. The investigations were carried out in accordance with the declaration of Helsinki and all patients gave their written informed consent for publication of the pseudonymized results.

In all patients the diagnosis of COVID-19 was confirmed by positive SARS-CoV-2 PCR from nasopharyngeal swab exams. All patients were severely affected and required mechanical ventilation, one of them also requiring veno-venous extracorporeal membrane oxygenation. Basic demographic and clinical characteristics are summarized in Table 1. All patients survived the disease and have been dismissed or transferred for neurological rehabilitation.

**Table 1:** Basic clinical, laboratory, and neurological characteristics of all four patients.

	**Patient 1**	**Patient 2**	**Patient 3**	**Patient 4**
**Gender**	**Male**	**Male**	**Male**	**Male**
Age [years]	70	68	71	29
BMI	24.8	27.8	23	38.5
SOFA score at ICU admission	13	6	12	11
APACHE II score at ICU admission	29	11	33	35
**Respiratory**				
- paO2/FiO2 (min) during ICU stay	59.40	85.6	131.8	81.00
- paO2/FiO2 (min) ICU admission	59.40	151.64	172.75	81.00
- paO2/FiO2 (min) day 1	198.18	150.17	147.25	112.66
- paO2/FiO2 (min) day 3	83.33	99.67	180.00	vvECMO
- paO2/FiO2 (min) day 7	238.00	205.00	131.80	vvECMO
Invasive Ventilation [Y/N]	Y	Y	Y	Y
vvECMO [Y/N]	N	N	N	Y
**Kidney**				
Creatinine max during ICU stay [mg/dl]	1.04	1.89	3.64	1.55
RRT [Y/N]	N	Y	Y	N
**Laboratory tests**				
Bilirubin max during ICU stay [mg/dl]	4.37	0.48	2.42	1.59
Ferritin max during ICU stay [μg/l]	1,263	1,647	2,668	3,215
D-Dimers at ICU admission [mg/l]	3.56	5.82	39.71	97.03
D-Dimers max during ICU stay [mg/l]	3.56	4.03	32.87	97.03
LDH max during ICU stay [U/l]	342	507	386	1,200
Troponin max during ICU stay [ng/l]	63	90	502	72
IL-6 (max) [pg/ml]	391.2	265.7	315.3	1,057
PCT (max) [ng/ml]	2.35	2.84	1.07	1.85
CRP (max) [mg/dl]	36.9	31.9	26.3	28.6
WBC (max) [×1,000/μl]	20.2	19.5	5.3	11.8
Lymphocyte count (min) [×1,000/μl]	0.86	0.93	0.33	0.94
**Neurological examination**				
Cranial nerves	Normal	**L: facial palsy, Bell sign** **+**	**Nystagmus**	Normal
Motor	**T2: tetraparesis**	**Tetraparesis**	**T1: no movements** **T2: tetraparesis**	**T1: tetraparesis** **T2: add. arm paresis left**
Atrophy	**T2:** **+**	**+**	T1: – **T2:** **+**	-
Tendon reflexes (L/R)	**↑↑/↑**	**–/–**	**T1:** **↑↑** **/** **↑**	**T1:** **↓/↓** **T2:** **↑/↑**
Babinski sign (L/R)	**T2:** **+/**–	–/–	–/–	–/–
Cloni (L/R)	**T1:** **+/+** T2: -/-	–/–	**T1:** **+/+** T2: –/–	T1: –/– **T2:** **+/+**
**Outcome**				
Length of ICU stay (days)	23	21	37	17
Length of hospital stay (days)	77	48	37	23
Hospital survival	Y	Y	Y	Y

*BMI, body mass index; SOFA, Sepsis-related organ failure assessment; APACHE, Acute Physiology And Chronic Health Evaluation; ICU, intensive care unit; Y, yes; N, no; vvECMO, veno-venous extracorporal membrane oxygenation; max, maximum; RRT, renal replacement therapy; LDH, lactate dehydrogenase; IL, Interleukin; PCT, procalcitonin; CRP, c-reactive protein; WBC, white blood cell; L/R, left/right; T1/T2, time of investigation. Pathological results of neurological examination are in boldface*.

**Figure 1:**
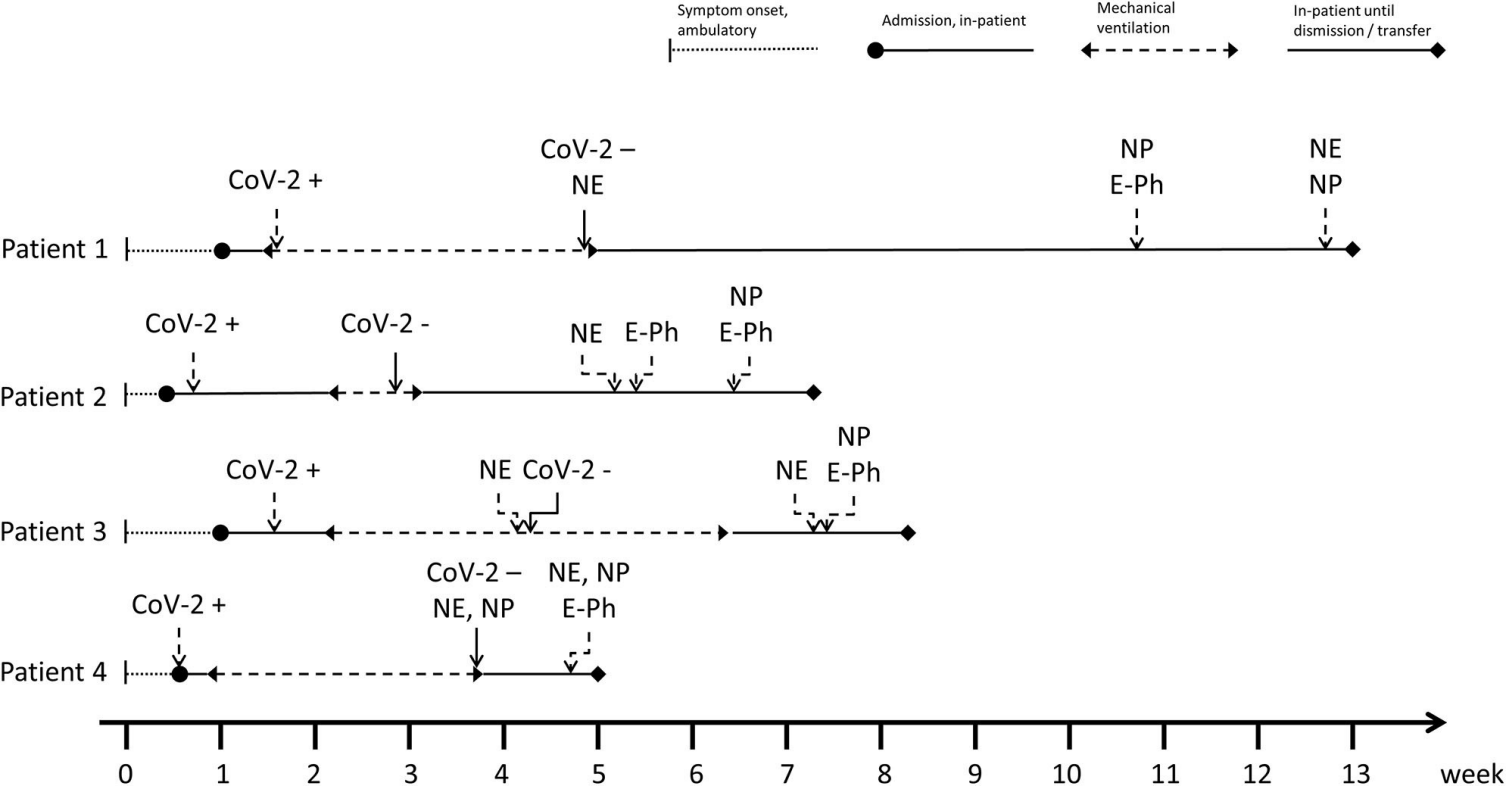
Detailed time course and series of events of all four patients, including the duration of mechanical ventilation, and time of neurological, neurophysiological, and neuropsychological investigations. CoV-2 +/–, CoV-2 PCR first time positive/negative; NE, neurological examination; NP, neuropsychological examination; E-Ph, electrphysiological invesitigations.

Neurological deficits were detected after cessation of sedation required for invasive ventilation and prone positioning and the patients regaining consciousness. Subsequently, intense neurological workup was initiated including multimodal neurophysiological and neuropsychological investigations. Patient 1 additionally suffered from intestinal ischemia requiring surgery. Therefore, neurological workup was performed later after full clinical recovery from surgery. All patients underwent either cerebral computed tomography or magnetic resonance imaging. Patient 1 and 2 underwent lumbar puncture for analysis of cerebrospinal fluid (CSF) including SARS-CoV-2 PCR. Figure 1 illustrates the detailed time course and series of events of all four patients, including the duration of mechanical ventilation and time of neurological, neurophysiological, and neuropsychological investigations.

### Neuropsychological Studies

Montreal Cognitive Assessment (MoCA) (15) and Symbol Digit Modality Test (SDMT) (16) were used to screen for cognitive impairment except in patient 3, who underwent Mini-Mental State Examination (MMSE) (17), since he was not able to complete the aforementioned tests due to cognitive dysfunction and severe attention deficits. In patient 1 and 4, follow up assessments were performed to evaluate the temporal dynamics. Rapid clinical test for delirium (4AT) was used to define the degree of possible acute delirium in all patients.

### Electrophysiological Studies

All patients underwent multimodal clinical neurophysiological measurements consisting of electroencephalography (EEG), nerve conduction studies (NCS), blink reflex (BR), sympathetic skin responses (SSR), electromyography (EMG), motor evoked potentials (MEP), and somatosensory evoked potentials (SEP) (18). We used a standard clinical neurophysiology device (Neuropack X1, Nihon Kohden, Japan) for all measurements. EEG used standard 10–20 system of scalp electrode placement. NCS included tibial, sural, and ulnar nerves and was extended as needed. BR of the bilateral orbicularis oculi muscle as measure for brain stem function and SSR were measured. Needle EMG were recorded from the tibialis anterior (TA), rectus femoris and first dorsal interosseous (FDI) muscles. Transcranial magnetic stimulation was applied via standard circular coil (MagPro compact, MagVenture, Germany) to record motor evoked potentials (MEP) from bilateral FDI and TA. The EMG signals were amplified, band passed between 5 Hz and 5 kHz and digitized at a sampling rate of 5 kHz. Sensory evoked potentials (SEP) used supramaximal stimuli of bilateral medial and tibial nerves with at least 200 averages.

## Results

### Neurology

Neurological examination (Table 1) revealed both affection of CNS and PNS in patients 1, 3 and 4. While patient 1 and 3 first showed signs for CNS affection and developed signs for PNS affection later on, it was the other way around in patient 4. Patient 2 presented with pure PNS affection similar to Guillain-Barré syndrome (GBS). Basic CSF analytics including cell count and protein level were unremarkable and PCR for neurotropic virus including SARS-CoV-2 were negative in both patients 1 and 2 who underwent lumbar puncture. In patient 2, who clinically presented with a GBS-like phenotype, antineuronal antibodies, and antiganglioside antibodies were negative as well. Cerebral imaging did not reveal any acute pathologies in all patients (Table 2). None of the patients reported hyposmia or ageusia.

**Table 2:** Detailed results of neuropsychological examination and multimodal electropysiological investigations.

**Investigation**	**Patient 1 (70 y)**	**Patient 2 (68 y)**	**Patient 3 (71 y)**	**Patient 4 (29 y)**
**EEG**				
Frequency	**Mild slowing (7 Hz)**	**Slowing (6 Hz)**	**Slowing (5 Hz)**	**Normal (10 Hz),** **fluctuation of vigilance**
Epileptiform activity	None	None	None	None
**MEP**				
First dorsal interosseus	n.a.^a^			
CMCT (ms)		**L: prolonged (9.2)** **R: prolonged (9.5)**	**L: prolonged (9.0)** R: normal (7.2)	L: normal (6.4) R: normal (7.2)
Amplitude (relative to CMAP)		L: normal (0.9) R: normal (0.61)	L: normal (3.2) R: normal (0.2)	L: normal (1.1) R: normal (0.67)
Tibialis anterior	n.a.^a^			
CMCT (ms)		n.a.^b^	n.a.^b^	L: normal (8.6) R: normal (9.0)
Amplitude (relative to CMAP)		L: normal (0.4) R: normal (0.72)	L: normal (2.76) R: normal (0.27)	L: normal (0.19) R: normal (0.50)
**SEP**				
Median nerve	n.a.^a^	n.a.^c^	n.a.^c^	
N20 latency (ms)				L: normal (8.6) R: normal (9.0)
N20-P25 amplitude (μV)				L: normal (0.19) R: normal (0.50)
Tibial nerve	n.a.^a^	n.a.^c^		
Latency P40 (ms)			**L: delayed (59.5)** **R: not reproducible**	L: normal (8.6) R: normal (9.0)
N30-P40 amplitude (μV)			**L: borderline (0.8)** **R: not reproducible**	L: normal (0.19) R: normal (0.50)
**BR**				
R1 (ms)	L: normal (10.5) R: normal (8.1)	**L: delayed (13.9)** R: normal (8.2)	L: normal (4.4) R: normal (4.4)	L: normal (11.4) R: normal (11.5)
iR2 (ms)	L: normal (28.4) R: normal (26.4)	L: normal (29.0) R: normal (30.0)	L: normal (27.4) R: normal (30.5)	L: normal (32.5) R: normal (32.6)
cR2 (ms)	L: normal (26.1) R: normal (26.4)	L: normal (30.3) R: normal (30.8)	L: normal (27.8) R: normal (30.5)	L: normal (34.9) R: normal (34.4)
**SSR^d^**				
Latency (s)	**palmar: delayed (2.3)** **plantar: delayed (2.7)**	**palmar: delayed (1.7)** **plantar: delayed (2.2)**	**palmar: delayed^e^** **plantar: delayed^e^**	**palmar: delayed (1.6)** **plantar: delayed (3.7)**
Amplitude (mV)	palmar: normal (0.7) plantar: normal (2.0)	palmar: normal (0.5) **plantar: reduced (0.2)**	**palmar: reduced^e^** **plantar: reduced^e^**	palmar: normal (0.4) plantar: normal (0.3)
**NCS^f^**				
**Tibial nerve**				
DML(ms)	Normal (4.2)	T1: Normal (4.4) T2: normal (4.3)	**Delayed (6.96)**	Normal (3.0)
mCV (ms)	Normal (44)	**T1: delayed (32.5)** **T2: delayed (32.7)**	**Delayed (32.1)**	Normal (48)
CMAP amplitude (mV)	**reduced (0.8)**	**T1: reduced (1.8)** **T2: reduced (3.9)**	**Reduced (0.72)**	Normal (13.7)
F-wave latency (ms)	**No response**	**T1: no response** **T2: no response**	**No response**	Normal (27.5)
Sural nerve				
SNAP amplitude (μV)	**No response**	**T1: reduced (1.8)** **T2: reduced (2.5)**	**No response**	Normal (7.2)
sCV (m/s)	**No response**	**T1: delayed (37.6)** **T2: delayed (32.1)**	**No response**	Normal (55)
Ulnar nerve				
DML (ms)	Normal (2.8)	T1: normal (3.1) T2: normal (3.2)	**Delayed (4.4)**	**Delayed (4.1)**
CMAP amplitude (mV)	Normal (8.5)	**T1: reduced (3.5)** **T2: reduced (3.1)**	**Reduced (0.8)**	**Reduced (1.8)**
mCV (m/s)	Normal (48)	**T1: delayed (42.2)** **T2: delayed (37.2)**	**Delayed (37.5)**	**Delayed (38)**
F-waves latency (ms)	**Delayed (33)**	**T1: no response** **T2: no response**	**No response**	Normal (31)
SNAP (μV)	**Reduced (3.5)**	**T1: no response** **T2: no response**	**No response**	**No response**
sCV (m/s)	**Delayed (40)**	**T1: no response** **T2: no response**	**No response**	**No response**
Median nerve	n.a.^a^	n.a.^a^	n.a.^a^	
DML (ms)				Normal (3.2)
CMAP amplitude (mV)				Normal (8.8)
mCV (m/s)				Normal (49)
F-waves latency (ms)				Normal (26.5)
SNAP (μV)				Normal (7.2)
sCV (m/s)				Normal (55)
**NCS^f^**				
Ulnar nerve	n.a.^a^	n.a.^a^	n.a.^a^	
DML (ms)				Normal (3.4)
CMAP amplitude (mV)				Normal (2.9)
mCV (m/s)				Normal (54.5)
F-waves latency (ms)				Not done
SNAP (μV)				Normal (7.9)
sCV (m/s)				Normal (75.6)
**EMG**				
Tibialis anterior	**Abnormal spontaneous activity**	**Abnormal spontaneous activity**	**Abnormal spontaneous activity**	n.a.^a^
Rectus femoris	Normal	**Abnormal spontaneous activity**	Normal	n.a.^a^
First dorsal interosseus	**Abnormal spontaneous activity**	**Abnormal spontaneous activity**	**Abnormal spontaneous activity**	**Abnormal spontaneous activity**
Abductor pollicis brevis	n.a.^a^	n.a.^a^	n.a.^a^	**Abnormal spontaneous activity**
Extensor digitorum communis	n.a.^a^	n.a.^a^	n.a.^a^	Normal
Deltoid				Normal
Imaging	cMRI + csMRI: normal	cCT: moderate microangiopathy	cCT: pre-existing cerebellar postischemic lesion	cCT: normal
**Neuropsychology^g^**				
MoCa (total score/30)	**T1: impaired (21)** **T1: impaired (21)**	**Impaired (16)**	n.a.^a^	**T1: not possible** **T1: impaired (21)**
MMSE (total score, /30)	T2: normal	n.a.^a^	**Impaired (14)**	n.a.^a^
SDMT (z-score)	**T1: impaired (-1.81)** **T2: impaired (-1.59)**	**Impaired (-4.29)**	n.a.^a^	**T1: not possible** **T2: impaired (-2.39)**
4AT (total score)	T1: normal (1) T2: normal (0)	Normal (2)	**Impaired (8)**	**T1: impaired (7)** T2: normal (1)

*Pathological results are in boldface*.

a *not available, investigation not performed*.

b *no calculation of CMCT possible due to missing root responses and F-waves*.

c *not possible due to insufficient relaxation*.

d *stimulation of the right N. medianus, recording left palmar/plantar*.

e *values missing due to technical reasons*.

f *NCS were repeated in patients 1 and 3 with an interval of 8 and 5 days between T1 and T2, respectively*.

g *neuropsychological tests were repeated in patients 1 and 4 with an interval of 13 and 16 days between T1 and T2, respectively*.

*T1/T2, time of investigation; L, left; R, right; EEG, electroencephalography; MEP, motor evoked potentials; CMCT, central motor conduction time; CMAP, compound motor action potential; SEP, somatosensory evoked potentials; BR, blink reflex; R, response; iR, ipsilareral response; cR, contralateral response; NCS, nerve conduction studies; DML, distal motor latency; mCV, motor conduction velocity; SNAP, senory nerve action potential; sCV, sensory conduction velocity; SSR, sympathetic skin response; EMG, electromyography; cMRI, cerebral magnetic resonance imaging; csMRI, cervicospinal magnetic resonance imaging; cCT, cerebral computed tomography; MoCA, Montreal Cognitive Assessment; MMSE, Mini-Mental State Examination; SDMT, Symbol Digit Modalities Test; 4AT, rapid clinical test for delirium*.

### Neuropsychology

All patients showed clinically relevant impairment of cognition (Table 2). Patients 1 and 2 had marked impairment of MoCA and SDMT despite lack of delirium. Patients 3 and 4 had signs for possible delirium in the initial 4AT. In patient 3, formal neuropsychological follow up was not possible due to dismission. However, 3 weeks after the initial examination, his relatives reported that he did not present signs of delirium anymore but still suffered from reduced psychomotor speed and alertness. In patient 4, after normalization of the 4AT score in the follow up 2.5 weeks later, cognition was still relevantly impaired.

### Electrophysiology

All patients showed signs of central nervous system affection. EEG showed diffuse slowing compatible with encephalopathy in all except for patient 4. No clear reaction to eye opening/closure or triphasic waves have been observed. Two patients had evidence of pyramidal tract affection on MEP, one of them additionally presented pontine brain stem affection evidenced by BR. In all patients, we further found affection of the peripheral nervous system measured by NCS and EMG. While three had polyneuropathy, patient 4 had signs of affection of the left brachial plexus. Moreover, all four patients had affection of the autonomic nervous system, measured by SSR. The detailed results of the multimodal neurophysiological studies are summarized in Table 2.

## Discussion

This case series provides direct *in vivo* evidence for functional brain stem involvement in COVID-19 patients. Moreover, sustained cognitive impairment may be frequent and independent of delirium in severely affected patients and requires particular attention since neuropsychological sequelae following COVID-19 could be of considerable social and socioeconomic relevance.

Experimental evidence from animal studies on SARS-CoV-1 and MERS-CoV have revealed the potential of nervous system affection by corona virus, suggesting a trans-synaptic transfer to the brain stem with consecutive spreading into the CNS (8–10, 13). Therefore, SARS-CoV-2 has also been suggested to be potentially neurotropic and possibly affecting the brain stem (8). It is also possible that CNS affection may be caused by a neuroimmune response triggered by the viral infection (19). Although a case with brain stem affection due to COVID-19 associated acute hemorrhagic brain stem lesion has recently been described (20), electrophysiologically proven brain stem affection without evident morphological changes has not been reported yet. To the best of our knowledge, our results include a single case report on patient 2 providing first direct electrophysiological evidence of functional brain stem involvement by BR in COVID-19. Consistently, affection of pontomedullary cardiorespiratory centers within the brain stem might promote severe course of the disease by contributing to the often clinically silent respiratory failure or weaning difficulties from invasive mechanical ventilation even after the recovery from pneumonia in COVID-19 (14). Another potentially useful method to investigate brain stem affection would be the monitoring of auditory brain stem reflexes and could be considered in future studies (13).

Another important observation is the potential of COVID-19 to result in clinically relevant and persistent cognitive impairment over weeks. We cannot rule out that initial cognitive impairment was at least partly due to delirium in these critically ill patients with a prolonged ICU stay. However, all patients had sustained cognitive impairment outlasting the acute phase of the disease for weeks when the 4AT score for delirium was unobtrusive. Further long-term follow up of cognitive function in a larger population of patients affected with COVID-19 is required, as in some patients, recovery of cognitive function may be a long lasting process. If full recovery of cognitive function should not be achieved, severe psychosocial and socioeconomic sequelae may result.

Of note, all patients studied here presented affection of the PNS as well. In all cases we found mixed axonal and demyelinating neuropathy affecting both sensory and motor nerves. Although we cannot fully exclude some degree of preexisting neuropathy, lack of neuropathy in the medical history and prominent abnormal spontaneous activity in EMG suggest an acute type of neuropathy in our case series. It has already been reported that COVID-19 may be associated with the occurrence of GBS or Miller Fischer syndrome (5, 21–23). In our cases, neurological deficits were detected 4 to 5 weeks after disease onset, which may be considered rather late for GBS but more likely to result from critical illness polyneuropathy (CIP). However, since all of our patients were severely affected requiring mechanical ventilation, it was difficult to determine the exact onset of neurological symptoms as sufficient neurological examination was not possible until they regained consciousness. Interestingly, all of our patients had electrophysiologically proven affection of the autonomic nervous system evidenced by SSR, which has been supposed to be rarely impaired in CIP but is much more common in GBS (24–26). Irrespective of the cause of PNS affection, autonomic dysfunction might also play a relevant role for cardiorespiratory failure in COVID-19 patients.

Finally, one of our patients presented with an additional monoparesis of his left arm, which was confirmed to be resulting from affection of the brachial plexus. Although we cannot rule out other causes like mechanical alteration during ICU stay with prone positioning, SARS-CoV-2 infection associated neuritis of the brachial plexus may also be the cause, given its neuroinvasive potential to affect the PNS.

There are some limitations which need to be taken into account. The main limitation is the small number of subjects that were investigated here. Moreover, this case series is based on analysis of data acquired during clinical patient care and is therefore without rigid study design, thus some missing values including CSF data could not be avoided. Also, a more elaborated neuropsychological testing would have been helpful. However, initial cognitive testing was performed in the ICU shortly after the end of mechanical ventilation. Therefore, a more detailed testing was difficult to perform in this setting. We also cannot completely rule out premorbid cognitive deficits since neuropsychological testing before COVID-19 have not been conducted in any of the patients. However, none of the patients had any difficulties on cognition or activities of daily living before COVID-19 supportive for premorbid cognitive decline. Moreover, two of the patients' follow up neuropsychological testing showed improvement in the course which supports the notion that the cognitive deficits were not preexisting.

Taken together, our results support the notion of SARS-CoV-2 related affection of the CNS and PNS. Neurologic impairment can be evidenced by an early and comprehensive clinical and electrophysiological workup. Early detection of neurological sequelae is important and may be of prognostic value, e.g., in the context of disabling peripheral neuropathy or affection of medullary cardiorespiratory centers possibly playing a role in the respiratory failure in patients with COVID-19. Thus, we recommend early screening for possible CNS and especially brain stem affection. As sustained cognitive impairments can occur and neuropsychological sequelae may be of particular psychosocial relevance, further long-term investigations on larger cohorts of severe cases are urgently warranted.

## Data Availability Statement

The raw data supporting the conclusions of this article will be made available by the authors, without undue reservation.

## Ethics Statement

Ethical review and approval was not required for the study on human participants in accordance with the local legislation and institutional requirements. The patients/participants provided their written informed consent to participate in this study. Written informed consent was obtained from the individual(s) for the publication of any potentially identifiable images or data included in this article.

## Author Contributions

SJG designed and conceptualized study, analyzed the data, drafted, and revised the manuscript for intellectual content. CB, SE, and TB acquired and analyzed the data and revised the manuscript for intellectual content. TM, DK-M, CV, TF, AK, B-EO, H-PH, and AS acquired data and revised the manuscript for intellectual content. PA designed and conceptualized study, analyzed the data, and revised the manuscript for intellectual content. All authors contributed to the article and approved the submitted version.

## Conflict of Interest

The authors declare that the research was conducted in the absence of any commercial or financial relationships that could be construed as a potential conflict of interest.

## References

[1] Guan W Ni Z Hu Y Liang W Ou C He J Clinical characteristics of coronavirus disease 2019 in China. N Engl J Med. (2020) 382:1708–20. 10.1056/NEJMoa2002032 32109013 PMC7092819

[2] Chen T Wu D Chen H Yan W Yang D Chen G. Clinical characteristics of 113 deceased patients with coronavirus disease 2019: retrospective study. BMJ. (2020) 368:m1091. 10.1136/bmj.m1091 32217556 PMC7190011

[3] Asadi-Pooya AA Simani L. Central nervous system manifestations of COVID-19: a systematic review. J Neurol Sci. (2020) 413:116832. 10.1016/j.jns.2020.116832 32299017 PMC7151535

[4] Mao L Jin H Wang M Hu Y Chen S He Q. Neurologic manifestations of hospitalized patients with coronavirus disease 2019 in Wuhan, China. JAMA Neurol. (2020) 77:683–90. 10.1001/jamaneurol.2020.1127 32275288 PMC7149362

[5] Toscano G Palmerini F Ravaglia S Ruiz L Invernizzi P Cuzzoni MG. Guillain-Barré syndrome associated with SARS-CoV-2. N Engl J Med. (2020) 382:2574–7. 10.1056/NEJMc2009191 32302082 PMC7182017

[6] Helms J Kremer S Merdji H Clere-Jehl R Schenck M Kummerlen C Neurologic features in severe SARS-COV-2 infection. N Engl J Med. (2020) 382:2268–70. 10.1056/NEJMc2008597 32294339 PMC7179967

[7] Hoffmann M Kleine-Weber H Schroeder S Krüger N Herrler T Erichsen S. SARS-CoV-2 cell entry depends on ACE2 and TMPRSS2 and is blocked by a clinically proven protease inhibitor. Cell. (2020) 181:271–80.e8. 10.1016/j.cell.2020.02.052 32142651 PMC7102627

[8] Li YC Bai WZ Hashikawa T. The neuroinvasive potential of SARS-CoV2 may play a role in the respiratory failure of COVID-19 patients. J Med Virol. (2020) 92:552–5. 10.1002/jmv.25728 32104915 PMC7228394

[9] McCray PB Pewe L Wohlford-Lenane C Hickey M Manzel L Shi L. Lethal infection of k18-hace2 mice infected with severe acute respiratory syndrome coronavirus. J Virol. (2007) 81:813–21. 10.1128/JVI.02012-06 17079315 PMC1797474

[10] Netland J Meyerholz DK Moore S Cassell M Perlman S. Severe acute respiratory syndrome coronavirus infection causes neuronal death in the absence of encephalitis in mice transgenic for human ACE2. J Virol. (2008) 82:7264–75. 10.1128/JVI.00737-08 18495771 PMC2493326

[11] Wu Y Xu X Chen Z Duan J Hashimoto K Yang L. Nervous system involvement after infection with COVID-19 and other coronaviruses. Brain Behav Immun. (2020) 87:18–22. 10.1016/j.bbi.2020.03.031 32240762 PMC7146689

[12] Matsuda K Park CH Sunden Y Kimura T Ochiai K Kida H The vagus nerve is one route of transneural invasion for intranasally inoculated influenza A virus in mice. Vet Pathol. (2004) 41:101–7. 10.1354/vp.41-2-101 15017022

[13] Ogier M Andéol G Sagui E Dal Bo G. How to detect and track chronic neurologic sequelae of COVID-19? Use of auditory brainstem responses and neuroimaging for long-term patient follow-up. Brain Behav Immun Heal. (2020) 5:100081. 10.1016/j.bbih.2020.100081 32427134 PMC7227537

[14] Baig AM. Computing the effects of SARS-CoV-2 on respiration regulatory mechanisms in COVID-19. ACS Chem Neurosci. (2020) 11:2416–21. 10.1021/acschemneuro.0c00349 32600045 PMC7422910

[15] Nasreddine ZS Phillips NA Bédirian V Charbonneau S Whitehead V Collin I The montreal cognitive assessment, MoCA: a brief screening tool for mild cognitive impairment. J Am Geriatr Soc. (2005) 53:695–9. 10.1111/j.1532-5415.2005.53221.x 15817019

[16] Smith A Symbol Digit Modalities Test. Los Angeles: West Psychology Services (1973).

[17] Folstein MF Folstein SE McHugh PR. “Mini-mental state.” A practical method for grading the cognitive state of patients for the clinician. J Psychiatr Res. (1975) 12:189–98. 10.1016/0022-3956(75)90026-6 1202204

[18] Levin K Lüders H Comprehensive Clinical Neurophysiology. Oxford: Elsevier LTD (2000).

[19] Yesilkaya UH Balcioglu YH. Neuroimmune correlates of the nervous system involvement of COVID-19: a commentary. J Clin Neurosci. (2020) 78:449–50. 10.1016/j.jocn.2020.05.056 32505431 PMC7250753

[20] Dixon L Varley J Gontsarova A Mallon D Tona F Muir D. COVID-19-related acute necrotizing encephalopathy with brain stem involvement in a patient with aplastic anemia. Neurol Neuroimmunol Neuroinflammation. (2020) 7:e789. 10.1212/NXI.0000000000000789 32457227 PMC7286661

[21] Gutiérrez-Ortiz C Méndez-Guerrero A Rodrigo-Rey S San Pedro-Murillo E Bermejo-Guerrero L Gordo-Mañas R Miller fisher syndrome and polyneuritis cranialis in COVID-19. Neurology. (2020) 95:e601–5. 10.1212/WNL.0000000000009619 32303650

[22] Zhao H Shen D Zhou H Liu J Chen S Guillain-Barré syndrome associated with SARS-CoV-2 infection: causality or coincidence? Lancet Neurol. (2020) 19:383–4. 10.1016/S1474-4422(20)30109-5 32246917 PMC7176927

[23] Abu-Rumeileh S Abdelhak A Foschi M Tumani H Otto M. Guillain-Barré syndrome spectrum associated with COVID-19: an up-to-date systematic review of 73 cases. J Neurol. (2020) 1:1. 10.1007/s00415-020-10124-x 32840686 PMC7445716

[24] Bolton C Thompson J Bernardi L Voll C Young GB. The cardiac R-R variation and sympathetic skin response in the intensive care unit. Can J Neurol Sci. (2007) 34:313–5. 10.1017/S0317167100006739 17803028

[25] Su XW Palka SV Rao RR Chen FS Brackney CR Cambi F. SARS-CoV-2-associated Guillain-Barré syndrome with dysautonomia. Muscle Nerve. (2020) 62:E48–9. 10.1002/mus.26988 32445201 PMC7283744

[26] Taly AB Arunodaya GR Rao S. Sympathetic skin response in Guillain-Barré syndrome. Clin Auton Res. (1995) 5:215–9. 10.1007/BF01824010 8520217

